# Relating therapy for voices (the R2V study): study protocol for a pilot randomized controlled trial

**DOI:** 10.1186/1745-6215-15-325

**Published:** 2014-08-16

**Authors:** Mark Hayward, Clara Strauss, Leanne Bogen-Johnston

**Affiliations:** School of Psychology, University of Sussex, BN1 9RH Brighton, UK; Research & Development Department, Sussex Partnership NHS Foundation Trust, Nevill Avenue, BN3 7HZ Hove, UK

**Keywords:** Auditory hallucinations, Voices, Interpersonal, Relationship, CBT, Psychosis

## Abstract

**Background:**

Evidence exists for the effectiveness of cognitive behaviour therapy for psychosis with moderate effect sizes, but the evidence for cognitive behaviour therapy specifically for distressing voices is less convincing. An alternative symptom-based approach may be warranted and a body of literature has explored distressing voices from an interpersonal perspective. This literature has informed the development of relating therapy and findings from a case series suggested that this intervention was acceptable to hearers and therapists.

**Methods/Design:**

An external pilot randomized controlled trial (RCT) comparing outcomes for 15 patients receiving 16 hours (weekly sessions of one hour) of relating therapy and their usual treatment with 15 patients receiving only their usual treatment. Participants will be assessed using questionnaires at baseline, 16 weeks (post-intervention), and 36 weeks (follow-up).

**Discussion:**

Expected outcomes will include a refined study protocol and an estimate of the effect size to inform the sample size of a definitive RCT. If evidence from a fully powered RCT suggests that relating therapy is effective, the therapy will extend the range of evidence-based psychological therapies available to people who hear distressing voices.

**Trial registration:**

Current Controlled Trials ISRCTN registration number 44114663. Registered on 13 June 2013.

## Background

The experience of hearing voices (verbal ‘auditory hallucinations’) is one of the prominent features of schizophrenia in current systems of diagnostic classification [1]. Voice hearing is reported to occur in approximately 70% of patients with this diagnosis [2], although the experience is also common in other mental health conditions such as post-traumatic stress disorder and borderline personality disorder [3]. National Institute for Health and Care Excellence (NICE) guidance in the UK recommends that psychological therapy in the form of cognitive behavioral therapy (CBT) should be offered to all patients who have a diagnosis of schizophrenia [4]. Although this is appropriate in the case of psychosis more broadly, where an evidence base exists for moderate effects [5], the impact of CBT for psychosis specifically on distressing voices is less convincing [6]. Therefore, consistent with a symptom-based approach [7], a voice-specific approach may be warranted. A recent review [6] was conducted of 16 published randomized controlled trials (RCTs) of CBT for psychosis that have reported at least one psychometrically validated outcome measure specifically related to voice hearing. The review found that the majority of studies failed to show a significant effect of CBT on voice hearing, and most of those that reported significant effects did so in the context of methodological weaknesses. Only one study reported an effect using a robust methodology. Interestingly, this was the only study that focused exclusively on voice hearing [8]. A factor that may have limited the effect of CBT upon voice hearing concerns outcome measurement - early trials used measures of voice frequency and severity, despite CBT not focusing upon the eradication of voices. The authors conclude by calling for more robustly designed RCTs of CBT aimed specifically at distressing voices. They draw attention to the potential for the integration of other psychotherapeutic techniques that have shown early promise for assisting voice hearers. In particular, the approach of trying to understand and adapt the interpersonal-like relationships that can develop with voices (see [9, 10] for a review).

There is an evolving body of literature that has explored the experience of hearing voices from an interpersonal perspective, examining the interaction that can occur between the hearer and the voice(s) that is heard. Findings suggest that hearers, regardless of diagnosis, can have integrated, personally coherent relationships with their voices [11]. Two studies using social rank theory found marked differentials of power and social rank between the hearer and the voice, which place the voice in a dominant position, and mirror perceptions of self in relation to significant social others [12, 13]. However, whilst there has been a focus on dominance, research has paid less attention to the role of intimacy and closeness within relationships with voices. If relationships with voices do mirror relationships in the social world, they are likely to be imbued with all the complexity and idiosyncrasy of social relationships. Relating theory [14] proposes that relating occurs on two axes: a vertical ‘power’ axis characterized at each polar end by ‘upper’ and ‘lower’ (analogous to power differentials), and a horizontal ‘proximity’ axis characterized at each polar end by ‘close’ and ‘distant’. Studies that have explored voice hearing experiences through the lens of relating theory report that distressing voices are perceived as relating dominantly and intrusively [15–17], and are responded to through distant relating from the hearer. Mirroring has also been found between proximity styles of relating to voices and those of the hearers within their social relationships [18].

Collectively, this body of research suggests that voices can be understood within interpersonal frameworks. This conceptualization has given rise to a new generation of therapeutic approaches that seek to modify the hearer-voice relationship. A pilot RCT [8] that looked at a therapeutic approach to address the power dynamic with voices that issue commands (cognitive therapy for command hallucinations [CTCH]) found significant reductions in compliance behaviour within the therapy group, and this finding has been replicated in a definitive trial [19]. A therapeutic focus upon closeness and intimacy has been developed by Hayward *et al*. [16] in the form of relating therapy (RT) which aims to re-balance the hearer-voice relationship with regard to both power and proximity. Findings from a case series suggested that RT was acceptable to hearers who experienced persistent and distressing voices [16, 20]. Consistent with the call for evaluations of effectiveness to be methodologically rigorous [6], RT should now be evaluated within an RCT design.

### Research objectives and hypotheses

The main objective of the study will be to inform the development of a phase III definitive trial [21]. Specifically, this external pilot RCT will establish recruitment, retention, and follow-up rates to the trial, assess levels of adherence with the treatment, and establish treatment effect size relative to treatment-as-usual. This information will be used to finalize the design of the therapy protocol and the study protocol for the phase III trial.

This external pilot RCT is, by definition, underpowered to detect statistically significant effects. Voice-related distress, rather than voice activity (such as loudness or frequency) or voice attributions (such as beliefs about the origin of voices), is the primary target of the therapy. Therefore, the primary hypothesis for the definitive trial is that RT added to treatment-as-usual will reduce the distress associated with voice hearing compared with treatment-as-usual on its own.

Secondary hypotheses for the definitive trial are that RT will lead to: 1) Reductions in voice-related distress relative to the control condition that will be maintained over at least three months; 2) Reductions in voice-related distress that will be mediated by improvements in relating to and/or by voices (improvements in relating defined as reductions in voice dominance and intrusiveness, and hearer distance - measured by the Voice and You [VAY]questionnaire); 3) Improvements in recovery (measured by the Choice of outcome in CBT for psychoses [CHOICE] questionnaire), relating to people in the social world (measured by the shorter version of the Persons Relating to Others Questionnaire [PROQ3]), and mood and anxiety (measured by the Hospital Anxiety and Depression Scale [HADS]); and 4) Improvements in relating to people in the social world will be associated with improvements in relating to and/or by voices (improvements in relating defined as reductions in voice dominance and intrusiveness, and hearer distance - measured by the Voice and You [VAY] questionnaire).

## Methods/Design

**Figure 1:**
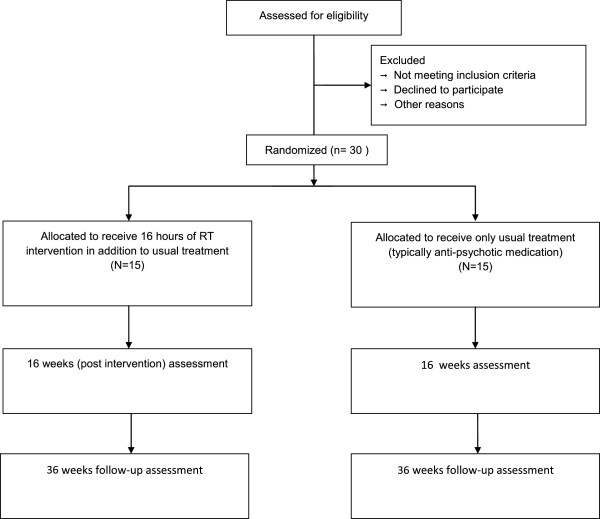
**Flow diagram for R2V trial.**

An external pilot RCT will compare the outcomes for patients receiving the RT intervention (in addition to their usual treatment) to outcomes for patients receiving only their usual treatment. Participants who consent will be randomized to either RT (plus treatment-as-usual) or to treatment-as-usual (TAU) only (see Figure 1). Randomization will be conducted by a statistician independent to the research team. Outcomes will be assessed at baseline (pre-randomization - Time 0), 16 weeks (post-intervention - Time 1), and 36 weeks (follow-up - Time 2). Time 1 and 2 assessments will be conducted blind by a researcher independent of the therapy process. Adherence to the therapy protocol will be assessed by an independent rater assessing a random selection of early, middle, and late recorded sessions using a modified version of the cognitive therapy checklist [22].

### Participants

The study aims to recruit approximately 30 patients from an NHS mental health trust in the south of the UK. Allowing for attrition from the study, there will be at least 12 completer participants per condition, in line with recommendations for pilot RCTs [22]. Inclusion criteria require that participants have been hearing distressing voices for at least one year, irrespective of diagnosis, and score 3 or above on either the intensity of distress item or the amount of distress item on the Psychotic Symptoms Rating Scale - Auditory Hallucinations Scale (PSYRATS-AHRS) [23] at the time of consent. People will be excluded on the grounds of organic illness, a primary diagnosis of substance misuse, or are currently receiving psychological therapy for distressing voices. Each participant will give informed consent before entering the trial.

### Planned intervention

The intervention will consist of a maximum of 16 hours (weekly sessions of one hour) of individual RT. The therapy protocol consists of three phases:

Phase 1: socialization to RT and its implications for the interrelating between hearer and voice. Use of chapter 3 from *Overcoming Distressing Voices* [24] to guide discussions about relationships in terms of power and proximity, and linking this discussion to participant’s experiences of relating to people and distressing voices. Consideration of the typical ways of responding to negative relating (giving in, fighting back, and trying to escape). Introduction of the possibility of relating differently to voices.

Phase 2: exploration of themes within the relational history of the hearer and their experience of relationships with voices, and interpersonal relating within the family and social environment (identifying any prominent themes, such as abuse, disempowerment, or rivalry). Development of connections across all forms of relating. If appropriate and desired, generating formulation(s) that link past and present forms of relating.

Phase 3: exploration and development of assertive approaches to relating (to the voice and socially). Selection of a relationship to be the focus of intervention. Use of chapter 7 from *Overcoming Distressing Voices* [24] to explore current utterances of chosen voice or person, responses to these utterances, identifying responses as passive, aggressive, or assertive and generation of assertive responses to chosen voice or other. Experiential role plays are a critical part of Phase 3 and are used to explore the motives of the voice (and other people) and to practice relating in an assertive manner.

The intervention will be offered by five therapists. Two of the therapists (including the first author) are familiar with the therapy, having previously delivered it within the case series [16, 20]. The three remaining therapists are experienced in the delivery of psychological therapy for psychotic experiences within trials, and will receive training from the first author.

### Treatment-as-usual

All participants will be receiving treatment-as-usual from an NHS mental health service. This will include anti-psychotic medication and regular contact with a member of their clinical team. Participants will be required to have no definite plan to receive psychological therapy for voices at the time of consenting to the study. There are no other requirements for previous or future therapy.

### Measures

#### Primary measure

Psychotic Symptoms Rating Scale (PSYRATS-AHRS) - an 11-item rating scale designed to measure the severity of different dimensions of the voice hearing experience [23]. Items include frequency, duration, loudness, intensity of distress, and controllability and are grouped together in four factors [25]; distress (negative content, distress, and control), frequency (frequency, duration, and disruption), attribution (location and origin of voices), and loudness (loudness item only). The authors report excellent psychometric properties [23].

#### Secondary measures

Choice of outcome in CBT for psychoses (CHOICE) – CHOICE is a 21-item self-report questionnaire developed with service users to assess goals for CBT for psychosis that are relevant to recovery, including self confidence, ways of dealing with unpleasant feelings and emotions, positive ways of relating to people, knowing they are not the only person who has unusual experiences, and a positive purpose and direction in life [26]. CHOICE has been found to be reliable and valid.

Voice and You (VAY) - the VAY is a 28-item measure of interrelating between the hearer and their predominant voice [15]. Relating is measured across four scales: two concerning the hearer’s perception of the relating of the voice (voice dominance and voice intrusiveness), and two concerning the relating of the hearer (hearer distance and hearer dependence). The VAY has good internal consistency (α >0.80 for all scales) and acceptable test-retest reliability (r >0.7 for all scales).

The Shorter version of the Persons Relating to Others Questionnaire (PROQ3) - the PROQ3 is a 40-item questionnaire that assesses relating across eight scales which correspond to the relating positions within Birtchnell’s Interpersonal Octagon [27]. The PROQ3 has acceptable internal consistency (α >0.70 for all scales) and its eight-factor structure is supported by factor analysis and multidimensional scaling analysis.

Hospital anxiety and depression scale (HADS) - the HADS is a 16-item measure of symptoms of anxiety and depression that has well-established psychometric properties [28].

### Analysis

The aim of this pilot study is to establish: 1) Recruitment, retention, and follow-up rates to the trial. This will be recorded as: (a) the number of research assistant hours required to obtain consent for one participant, (b) the percentage of participants who complete the Time 1 assessment, and (c) the percentage of participants who complete the Time 2 assessment; 2) Level of adherence with the treatment. This will be recorded as the percentage of participants who complete at least eight RT sessions (50% of the maximum of 16 sessions); 3) Treatment effect size relative to treatment-as-usual. A mixed one way analysis of variance (ANOVA) with post-hoc tests, where appropriate, will be conducted in order to calculate the group (two levels: RT + TAU or TAU) by time (three levels: T0, T1 and T2), interaction effect size, and its 95% confidence interval on the PSYRATS-AHRS; 4) Minimally clinically important difference. Taking a reduction of 1.0 on a PSYRATS-AHRS item as having clinical meaning, a difference of 5 in change scores (T0-T1 and T0-T2) between the two groups for the 5-item distress subscale will represent a minimally clinically important difference and be contained within the 95% confidence interval for the effect size.

This information will be used to refine the therapy protocol and to design the definitive trial. The group by time effect size on the PSYRATS-AHRS will be used for the power calculation for the definitive trial whilst taking into account the minimum clinically important difference on the PSYRATS-AHRS.

### Research governance

The study is sponsored by Sussex Partnership NHS Foundation Trust. NHS ethics (reference number 12/LO/2045) and R&D approval were sought before the commencement of the trial. Medical Research Council Guidelines on Good Clinical Practice in Clinical Trials [29] informed the constitution of the Trial Steering Committee, which includes an independent chair, two independent experts, and a lay person.

## Discussion

Evidence for the effectiveness of CBT specifically for distressing voices is limited and the development of alternative interventions is warranted [6]. We have contributed to the development of a literature that explores the experience of hearing voices within interpersonal frameworks. Specifically, we have explored voice hearing with respect to both the power and proximity aspects of relating and developed RT in order to specifically target these forms of relating. RT aims to re-balance the hearer-voice relationship (without extremes of power or proximal ways of relating) and has been found to be safe, acceptable, and intuitively appealing to therapists and hearers within a previous study [16, 20]. This external pilot RCT will generate a refined study protocol, an indication of recruitment and retention rates, and an estimate of the effect size, in order to inform the sample size calculation for a phase III trial. If evidence from a definitive RCT suggests that RT is effective, this will extend the range of evidence-based psychological therapies available to people who hear distressing voices.

## Trial status

Recruitment to the trial commenced in June 2013. At present, recruitment and data collection will continue until February 2015.

## Abbreviations

CBT: Cognitive behavioral therapy
CTCH: Cognitive therapy for command hallucinations
NICE: National institute for health and care excellence
RCT: Randomized controlled trial
RT: Relating therapy
TAU: Treatment-as-usual.
